# Virus–Host Interactions in Foot-and-Mouth Disease Virus Infection

**DOI:** 10.3389/fimmu.2021.571509

**Published:** 2021-02-26

**Authors:** Kangli Li, Congcong Wang, Fan Yang, Weijun Cao, Zixiang Zhu, Haixue Zheng

**Affiliations:** State Key Laboratory of Veterinary Etiological Biology, National Foot and Mouth Diseases Reference Laboratory, Key Laboratory of Animal Virology of Ministry of Agriculture, Lanzhou Veterinary Research Institute, Chinese Academy of Agricultural Sciences, Lanzhou, China

**Keywords:** FMDV, interactions, immune dysfunction, pathogenesis, viral infection

## Abstract

Foot-and-mouth disease (FMD) is a highly contagious disease of cloven-hoofed animals, which has been regarded as a persistent challenge for the livestock industry in many countries. Foot-and-mouth disease virus (FMDV) is the etiological agent of FMD that can spread rapidly by direct and indirect transmission. FMDV is internalized into host cell by the interaction between FMDV capsid proteins and cellular receptors. When the virus invades into the cells, the host antiviral system is quickly activated to suppress the replication of the virus and remove the virus. To retain fitness and host adaptation, various viruses have evolved multiple elegant strategies to manipulate host machine and circumvent the host antiviral responses. Therefore, identification of virus-host interactions is critical for understanding the host defense against virus infections and the pathogenesis of the viral infectious diseases. This review elaborates on the virus-host interactions during FMDV infection to summarize the pathogenic mechanisms of FMD, and we hope it can provide insights for designing effective vaccines or drugs to prevent and control the spread of FMD and other diseases caused by picornaviruses.

## Introduction

Foot-and-mouth disease (FMD) is an acute and highly contagious disease caused by foot-and-mouth disease virus (FMDV). FMDV has a broad host range, and the susceptible animals contain more than 70 cloven-hoofed animals, including pigs, cattle, sheep, goat and African buffaloes (1, 2). The clinical symptoms of FMD include fever, lameness and vesicular lesions on the feet, tongue and teats (3). FMDV is thought to spread mainly from animal to animal by aerosol droplets between animals in close contact. FMD seriously affects the livestock industry and threatens the international trade in animals and animal products. Therefore, it has been classified into the A list of infectious diseases of animals by the Office International des Epizooties (OIE) (4, 5).

**Figure 1:**
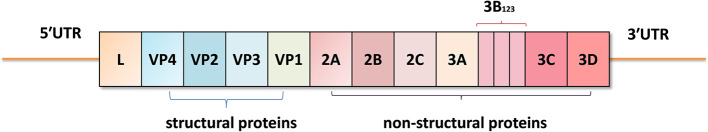
The viral genome structure of foot-and-mouth disease virus (FMDV). The viral genome contains a 5′-untranslated region (5′UTR), a large open reading frame (ORF) including the L, VP4, VP2, VP3, VP1, 2A, 2B, 2C, 3A, 3B(3B1, 3B2, and 3B3), 3C, and 3D coding regions, and a 3′UTR.

Foot-and-mouth disease virus (FMDV) belongs to the genus *Aphthovirus* within the family *Picornaviridae* (2). The viral genome is a single-stranded positive-sense RNA, approximately 8.3 kb in length, including a long 5′-untranslated region (5′UTR), a large open reading frame (ORF), and a short 3′UTR. The viral genome encodes four structural proteins VP1, VP2, VP3 and VP4 (also known as 1D, 1B, 1C and 1A) which constitute the icosahedral capsid, and eight non-structural proteins (L^pro^, 2A, 2B, 2C, 3A, 3B, 3C^pro^, 3D^pol^) that regulate RNA replication, protein folding and virus assembly (6) (**Figure 1**). FMDV has seven serotypes: O, A, C, SAT1, SAT2, SAT3, and Asia 1 (2). There is no effective cross-protection between different serotypes, which makes the prevention and control of FMD more difficult.

An immunosuppressive stage has been reported during the acute infection of FMDV in swine (7, 8). The immunosuppression and virulence of viral proteins efficiently promote FMDV replication which also affect the host’s resistance to other pathogens. Therefore, FMDV is an important pathogen that threatens the health of livestock. Two of our previous review papers have summarized how FMDV disrupts host RIG-I-like receptors pathway and type I interferon signaling (9, 10). As for this review, we focused on the pathogenesis of FMD, FMDV receptors and cell tropism, innate/adaptive immune system dysfunction (how FMDV causes immune cell dysfunction), autophagy, apoptosis and Golgi-endoplasmic reticulum pathways in FMDV infection. Meanwhile, we summarized how host defends FMDV infection through various host restriction factors. This will help clarify the pathogenesis of FMD and summarize the functions of viral proteins, and provide insights for designing effective vaccines and drugs to prevent and control the spreading of FMD.

## Pathogenesis of FMD

FMDV has multiple serotypes and broad host range (2). The clinical symptoms, pathogenesis and immune response vary with the hosts and serotypes. The pharyngeal region is the site for early localization and growth of FMDV in cattle and pigs, regardless of the infection methods and the serotypes of the virus (11, 12).

In the cattle infected by FMDV using aerosol infection, the virus develops a primary infection in the pharyngeal epithelium, and then replicates extensively in pneumocytes in the lungs (13). The virus starts to multiply in the epithelial cells at the beginning of the invasion in the cattle. After 1 to 2 days postinfection (dpi), the virus enters into the blood and spreads to different organs and tissues for secondary replication, resulting in clear viremia (14). The pharyngeal epithelium is also highly associated with the viral persistence in cattle (15). Therefore, how to eradicate the virus at the beginning of the invasion (in the pharyngeal epithelium) is critical for limiting the rapid spread of FMDV. Development of antiviral drugs targeting the pharyngeal epithelium might be a prominent strategy to control and prevent FMD. Single-cell analysis of the mainly infected cells in these tissues is also critical for clarification of the primary and secondary replication sites for FMDV.

The most common manifestation in FMDV-infected animals includes fever and lameness, and the vesicles in the mouth and feet can also be observed. A large number of viruses can be detected from the secretions, excreta, and tissues. At 3 to 4 dpi, the vesicles can be observed on the nipples, toes, and other hairless areas of the diseased cattle (16), and more vesicular lesions can be seen in the mouth, pharynx and nose of the animals, resulting in difficulty in swallowing and drooling (17). The generated vesicles are related to intense edema of the dermis and dense inflammatory infiltrate (18–20). The amount of NK cells producing IFN-γ transiently increases after 24 h of FMDV infection in swine, but rapidly declines at 2 dpi. Meanwhile, the viremia and fever reach its peak, the lymphopenia occurs, and the vesicular lesions are observed on the feet and snout (21). The specific mechanisms involved in this pathogenic process remain unknown. How the virus regulates the immune reaction and contributes to viral replication in the targeted tissues should be extensively explored, which might provide insights for development of drugs against FMDV.

In FMDV-infected animals, a series of immune responses are gradually induced to defend the host against the infection and suppress viral replication (22, 23). The virus-specific antibodies can be detected at 3 to 4 dpi in cattle. IgM increases significantly on the 5th day, and the IgG increases gradually, reaching the highest level at the 9th dpi (24). The virus can be removed from the body by the immune system within two weeks. However, the virus has evolved elegant strategies to counteract the immune system. In certain cases, the virus hides from the immune system and stores itself in the nasopharynx site, forming a persistent infection in the animals (25). In addition, FMDV also damages the immune cells and inhibits the immune signal transduction, leading to the dysfunction of immune system and quick replication of the virus (26–29). Many viral proteins have been identified to participate in disruption of host immune response, including both structural proteins and nonstructural proteins (9, 10). Innate immunity is vital for guiding the adaptive immune response (30–32). The innate and adaptive immune systems provide the complete line of defense against virus invasion (33, 34). Therefore, modification of the critical sites responsible for immunosuppressive effect will be helpful for improve the efficiency of vaccines and generation of modified viruses with low pathogenicity. Especially for the structural proteins, alleviation of the immunosuppressive effect of the antigen will accelerate and enhance the immune response in the vaccinated animals. Meanwhile, decreasing the pathogenicity of the vaccine strain by the reverse genetics will decrease the potential spreading risk of the virus after unexpected leakage of the virus.

## FMDV Receptors and Cell Tropism

Viruses bind to specific cell surface receptors and subsequently enter into host cells. The reported FMDV receptors include the integrin receptors (35), the heparan sulfate (HS) receptor (36), and the third receptor that has not been identified (37). FMDV attaches to host cells by recognizing these receptors on the cell surface, then gain entry into the host cells through receptor-mediated endocytosis. The specificity of virus-host interaction determines the host range and cell tropism, and the receptor pathway used by the virus is decisive for the invasive efficiency (38, 39). During the longer periods of virus-host co-evolution, amino acid sequence of the receptor binding site may occur mutations, leading to the change of the infection ability or invasive manners of the virus. Most of the field FMDV strains (especially for serotype O), uses the integrin receptors to enter into the host’s cell (40, 41). However, during the adaptation of field strains into the cell culture system, the virus can use the HS receptors and/or other unidentified receptors (42, 43). For instance, FMDV type O1 Campos variant including the VP3 Arg56 mutation can infect MCF10A cells by binding to HS receptor (44). Therefore, it is of great significance to understand the invasion mechanisms of the virus to prevent the spreading of FMD. Generation of premature termination codon–harboring viruses in the transgenic cell line containing orthogonal translation machinery is a prominent way for development of influenza A viruses (45). These studies imply that generation of vaccine strains that only replicate in some modified cells will decrease the potential threat of lab/factory facility leak of the virus.

Integrin is a kind of heterodimeric transmembrane glycoproteins formed by the non-covalently linked α and β subunits (46). It includes an extracellular domain, a transmembrane domain, and a cytoplasmic domain. RGD is a highly conserved tripeptide motif composed of arginine-glycine-aspartate (Arg-Gly-Asp), located in the G-H loop of FMDV structural protein VP1 (47). FMDV VP1 interacts with the integrin receptor through its RGD motif to mediate the initiation of viral infection. There are 24 integrin receptors that have been identified up to now, while only the αvβ1, αvβ3, αvβ6, αvβ8 act as FMDV receptors (48), and the αvβ3 and αvβ6 integrins are considered as the main receptors of FMDV. αvβ3 is the first known receptor for FMDV, which is preferentially expressed in the endothelial cells. αvβ6 is widely found in the epithelial cells of FMDV target tissues. This is also consistent with the fact that epithelial cells are often targeted by FMDV during the infection (40). Therefore, the αvβ6 integrin expressing cell-lines are beneficial for quick isolation of field strains in the cell culture system. A recent study has also identified a new FMDV receptor, J Jumonji C-domain Containing Protein 6 (JMJD6), which interacts with the mutated VP1 protein. When a specific mutation is introduced into VP1 protein, the C-terminus of JMJD6 will interact with that VP1 and initiate the infection of FMDV in CHO cells (49). However, whether JMJD6 is also efficiently used by different serotypes of FMDV in host cells remain unknown. Clarification of how FMDV enters into host cell will provide insight for developing effective drugs to block FMDV infection. The relation of JMJD6 with other viral receptors should also be explored to determine the main receptors responsible for FMDV entry in its natural hosts.

After the binding of FMDV VP1 protein with the integrin receptor, the specific amino acid motif within the receptor intracellular region interacts with the intracellular junction protein, which in turn transmits the internalization signal into the cell (50). The clathrin in the cytoplasm assembles to form clathrin-coated pit (CCP), and the CCP dissociates from the cell membrane and promote the formation of clathrin-coated vesicles, which deliver the virion into endosomes. Macropinocytosis is another endocytic pathway apart from the clathrin-mediated pathway (30). This explains why blocking the clathrin-mediated endocytosis pathway could only partly inhibit FMDV replication. After the internalization event, the uncoating rapidly occurs within the acidified endosomes. Finally, the viral RNAs are released into the cytoplasm by an undefined mechanism (51–53). Currently, few drugs targeting picornaviruses are available. Intervention of the uncoating process is also a strategy to inhibit the replication of the virus in host, and it is important to identify the viral domains participate into the internalization and uncoating during viral infection (54).

HS is a mucopolysaccharide located on the cell surface (55). It also acts as a receptor of FMDV for entering several host cells (44). Under physiological conditions, the N-sulfate group or the O-sulfate group in the HS carbon chain makes the sugar chain negatively charged. Therefore, the negatively-charged HS can undergo direct electrostatic adsorption with the positively-charged Arg56 within the VP3 of FMDV (56). FMDV binds to HS and then enters into host cells through the endocytic pathway. However, the HS-mediated endocytic rate is slower than that mediated by integrins, and the involved mechanisms are also different. When FMDV enters into the cells through HS, the virion will fall into the caveola and enter into the cytoplasm, then subsequently move to the recycling endosome, releasing the viral genomic RNA (44). JMJD6 contributes to FMDV infectivity and may be a potential receptor of FMDV in CHO cells. However, mutation analysis studies about the cell-adapted FMDV related to the JMJD6 receptor are still limited, and the pathways used by the virus after the internalization of FMDV remains unknown. The association of HS and JMJD6 should also be investigated.

In addition to utilize integrins and HS as FMDV receptors, there is a third group of receptors. FMDV infects macrophages through Fc receptor-mediated adsorption (57). The mutation of RGD to RDD, RSD, REG, SGD, RGG or GGG in VP1 retains the infectivity of FMDV (58–61). However, the receptors used by these mutants remain unknown. Therefore, it is commonly believed that a third group of receptors involved in the adsorption and internalization of FMDV exist in various host cells (62, 63). An immunoprecipitation assay combined with a Mass spectrometry method might be a useful tool to identify new members of the third group of receptors of FMDV. Comparison of the structural protein variations among viruses with different pathogenicity might also provide some clues for investigation of FMDV receptors.

## Innate/Adaptive Immune System Dysfunction Caused by FMDV Infection

Innate immunity is the first line of defense against viral infections. The innate immune system triggers a series of signal transduction cascades after sensing of the virus invasion, inducing the production of interferons (IFNs) and pro-inflammatory cytokines (64). IFNs activate the downstream signaling pathway through autocrine and paracrine, and promote the synthesis of a large number of IFN-inducible proteins, interfering with the synthesis of viral proteins and replication of the virus (65). Pro-inflammatory cytokines and chemokines rapidly activate and recruit natural immune cells to reach to the infection sites, and eliminate the invading viruses by phagocytosis or lysis (66). Meanwhile, the innate immune system further activates the adaptive immune system to induce the production of specific antibodies that eventually remove the viruses from the host (67). However, in order to survive and reproduce in the host, there are always a few of viruses can break through the innate immune barrier and block the activation of adaptive immune system (68). A series of antagonistic strategies to evade host immune responses have been established during the long-term evolution, which mainly include destruction of cell-mediated immune response, hiding of viral components and manipulation of cell machines (69, 70).

### Immune Cell Dysfunction Caused by FMDV Infection

Immune cells refer to the cells that participate in antiviral immune responses, which mainly contain the natural killer (NK) cells, dendritic cells (DCs), gamma delta T lymphocytes (γδ T cells), B lymphocytes, T lymphocytes, macrophages and granulocytes (71). Pattern recognition receptors (PRRs) are special molecules of host cells that are responsible for recognition of viral elements after virus invasion. Most of immune cells express abundant PRRs (72). Pathogen-associated molecular patterns (PAMPs) are the specific viral molecules targeted by PRRs. The activation of the innate immune pathways and the intensity of the immune responses are all related to the recognition of PAMPs by PRRs (73). Therefore, the mutual interaction between PRRs and PAMPs is critical for initiation of the immune response. When the immune responses are activated, the replication of the invaded virus will be suppressed (74). To counteract the antiviral response mediated by the immune cells, the invaded viruses have developed several effective strategies to cause the dysfunction of the immune cells and impair the involved antiviral response (75). Impairment of the immunosuppressive function of FMDV in these processes will help development of new drugs or vaccines against FMDV.

#### Macrophages-, NK Cells-, and DCs-Related Innate Immune Dysfunction

**Figure 2:**
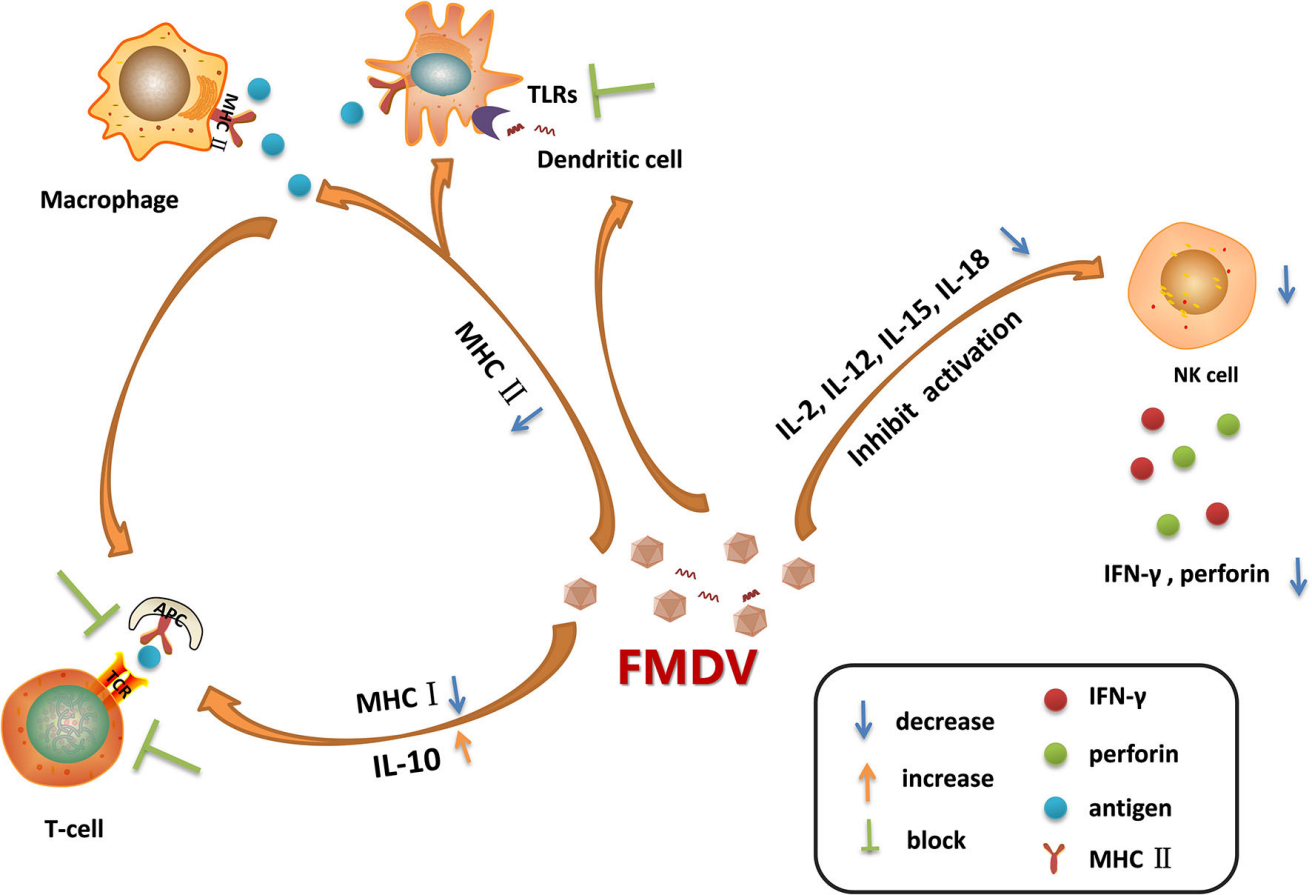
Immune cell dysfunction caused by foot-and-mouth disease virus (FMDV) infection. FMDV infection blocks the activation of Toll-like receptors (TLRs) pathways in dendritic cells. It also reduces the expression of MHC II molecules on the surface of dendritic cells and macrophages, leading to the decreased antigen presentation activity, which in turn blocking the activation of T cells. Moreover, FMDV infection induces the secretion of IL-10, and reduces the expression of MHC I molecules, suppressing the adaptive immune response mediated by T cells. As infection progresses, FMDV damages the cells that secrete IL-2, IL-12, IL-15, and IL-18, thus limiting NK cell activation and inhibiting the secretion of IFN-γ and perforin. The dysfunction of these immune cells during viral infection provides a favorable environment for multiplication of progeny virus.

After the invasion of FMDV into its host, macrophages are quickly recruited to the infection site, and the virions are ingested into the cells, secreting interleukin (IL)-12 and TNF (76). The NK cells are subsequently activated and secrete IFN-γ, which further activates macrophages and enhances its killing effect (77). Therefore, FMDV, FMDV-antibody complex, and the infected cells can be internalized and cleared under the action of lysosomes within the macrophages. In addition, the macrophages are used as antigen-presenting cells to deliver antigens to CD4+/CD8+T cells through MHC molecules (78). An MHC-restricted CD8^+^ T cell response is proved being induced in cattle by FMDV infection and also following treated with inactivated FMDV (79). However, FMDV could interfere with antigen presentation by decreasing the expression of MHC II on DC and monocytes through regulation of mucosal macrophages which may defer the DCs-mediated antiviral response (**Figure 2**) (80). FMDV infection downregulates the expression of MHC II and CD1 in porcine monocyte-derived DCs (Mo-DCs) (81). The expression of MHC II and CD40 molecules are also downregulated in FMDV-infected murine DCs (29). In these infected DCs, the expression of IL-10 is highly upregulated. Acute infection of pigs with FMDV also drastically increases IL-10 production by DCs, and the increment in the production of IL-10 impairs activation of T cells (**Figure 2**) (7). The suppression of immune function is directly associated with the viremia level. The viral proteins responsible for reducing the expression of MHC II molecule and induction of IL-10 production, and the involved mechanisms remain unknown. Further elucidation of how FMDV decreases MHC II molecule and induces IL-10 production will provide new insights for making new strategy to limit the replication of the virus. IL-6 is determined to be involved in the pathology of SARS-CoV-2 and COVID-19. Therefore, IL-6 has been determined to be an important target for treatment of COVID-19, and the IL-6 inhibitors are potential drugs against the virus infection (82, 83). The role of IL-10 inhibitors in treatment of FMD should also be explored which might help for control or eradication of FMDV.

NK cells kill the virus-infected cells without antigen stimulation, so they are named as natural killer cells (84). After FMDV invasion, NK cells can be activated by cytokines such as IL-2, IL-12, IL-15, IL-18, and IFNs, the activated NK cells then release a large amount of perforin and granzyme to kill the infected cells (26). NK cells also kill the target cells by binding to the Fc receptors of antibodies and release perforin and granzyme to directly kill the antibody-binding cells, this process is called antibody-dependent cell-mediated cytotoxicity (ADCC) (85). Moreover, NK cells induce the apoptosis of the infected cells through membrane binding receptors. The FASL and TRAIL ligands on NK cell membrane bind to Fas and apoptotic receptors (DRs) on the membrane of infected cells to activate caspases and induce a cascade of signaling to promote cell apoptosis (86). Meanwhile, NK cells regulate the activation of adaptive immune response by supporting the Th1 polarization. The cytokines produced by Th1 mainly enhance the inflammatory response and activate the T-cell immune response and macrophages (87). In the early stage of viral infection, NK cells produce high levels of IFN-γ and induce the antiviral effect. However, in FMDV-infected pigs, as the infection progresses, the NK cells become dysfunctional. In acute infection stages, the killing of viral infected cells was suppressed, the secretion of IFN-γ was reduced, and the capability of NK cells to secrete cytokines or store cytolytic molecules was inhibited as well (21). FMDV infection reduces the expression of IFN-γ and impairs the cytotoxicity of NK cells by blocking the signal transduction in the immune cells (**Figure 2**). VP3 has been reported to inhibit IFN-γ production during FMDV infection (88). The L^pro^ is determined to be a critical viral factor to suppress NK cell activity. The viruses infect and damage the cells that express IL-2, IL-12, IL-15, IL-18, and IFNs which are required for NK cells activation. L^pro^ blocks the activation of RLR pathway and inhibits the expression of various cytokines (89, 90). Meanwhile, L^pro^ cleaves eIF4G which also decreases the expression of IFNs and cytokines. This suggested that L^pro^ might be responsible for impairing the immune signal pathway activation in immune cells. NK cells express many receptors that direct the activity of NK cells during viral infection. However, the cytokine receptor mRNA expression pattern during FMDV infection has been showed having no correlation with the dysfunctional status of NK cells (21). Together, FMDV infection causes the destruction of these cells, resulting in the insufficient activation of NK cells, and benefits the replication of FMDV.

DCs play important roles in antigen presentation, which professionally deliver antigens to lymphocytes to induce immune response (91). DCs also secrete several cytokines for immune regulation. During the acute infection phase, FMDV infection stimulates DCs to secrete IL-10, leading to activation of humoral immunity and inducing effective neutralizing antibody response for viral clearance (7). However, as the infection progresses, the maturation of DCs and the ability of DCs to deliver antigens can be inhibited by FMDV. The TLRs signaling is also inhibited by FMDV in DCs (92), thus delaying the initiation of innate immune response (**Figure 2**). TLR3 and TLR7 are involved in RNA virus recognition. Hepatitis A virus, a human picornavirus, disrupts TLR3 signaling through cleavage of immune adaptor molecule TRIF by 3CD protein (93). However, it remains unknown how FMDV interfere with the TLR3 signaling. FMDV and immune complexes activate DCs through the TLR7 pathway (94). Whether there are viral proteins play antagonistic function against TLR7 to block type I IFN and inflammatory cytokines production in DCs during FMDV infection remains to be determined. Plasmacytoid DCs (pDCs) recognize FMDV-immunoglobulin complex by the FcγRII receptors (95), and play an important role in suppression of viral infection through induction of type I and type III IFN production. Circulating pDCs numbers and *in-vitro* pDCs IFN-α production are transiently decreased by 48 h following FMDV infection, suggesting an antagonistic effect of FMDV against the function of pDCs (96). Monocyte-derived DCs (MoDCs) also secrete type I IFNs. However, in FMDV-infected swine MoDCs, there is almost no IFN-α expression can be detected (92). Langerhans cells (LCs) are distributed in the epidermis. Porcine LCs can be absorbed by FMDV *in vitro* that results in the internalization of the virus into LCs, and subsequently triggers the secretion of IFN-α (97). However, in FMDV-infected pigs, LCs only secrete extremely low level of IFN-α. Conventional DCs (cDCs) are sentinel cells that can capture, process, and present antigens, inducing T cell activation and proliferation. While, in cattle cDCs, FMDV infection induces IL-10 secretion and causes the down-regulation of MHC II molecule, leading to the impaired virus clearance efficiency (80). MHC II is important in initiating immune responses. It plays multiple roles in inducing protective immunity to vaccination (98–100). Clarification of the regulatory mechanisms of MHC II during FMDV infection will help for optimization of FMDV MHC class II vaccine formulations. The distinctions between cattle and pigs in FMD pathogenesis events have been reported (17). Many factors have contributed to the different manifestation, which include variations in the routes of virus exposure, variations in the quantities of virus shed by aerogenous routes, and the capability of long-term persistence of infectious virus in tissues of ruminants, but not pigs. The different mechanisms used by the virus to interfere with the immune cells in pig and cattle might be a critical factor as well.

#### Dysregulation of T Cells and B Cells

T cells and B cells are critical for initiation of adaptive immune response, which has the characteristics of specificity, diversity, immune memory, self-recognition and non-self-recognition (101). T cell receptor (TCR), an antigen receptor on the cell membrane of T cells, recognizes antigens that bind to its own MHC molecules. T cells can be divided into three categories according to their functions: cytotoxic T cells (Tc), helper T cells (Th), and suppressor T cells (Ts) (102). After the viral infection, the processed viral peptides bind to MHC I molecule, the antigen-presenting cells are recognized by Tc cells, invoking perforin production that directly kill the pathogens and the infected cells (103). Similarly, there is an antigen receptor, B cell receptor (BCR) on the surface of mature B cells. Some of the B cells stimulated by the antigen rapidly proliferate and differentiate into plasma cells and secrete antibodies to specifically clear the antigen, while others will differentiate into memory cells to participate in the secondary immune response (104).

The rapid multiplication of FMDV in host cell could result in the decreased level of MHC I molecules, and the killing function of Tc cells will be deterred (21). The amounts of lymphocytes are significantly reduced as well. Although the numbers of lymphocytes recover rapidly at the 4th dpi, the functional defects of the T cells remain unresolved until the 7th day after infection. It suggests that there is a transient state of immunosuppression at the early infection of FMDV, which effectively promotes the viral reproduction (8, 105). Vaccination of FMDV vaccine often give rise to a rising titers of antibody (106). Whether the dysfunction of T cells has mainly contributed to the delayed immune response should be investigated. Moreover, the increased secretion of IL-10 by cDCs during FMDV infection also leads to the decrease T cell activity (7), and the amounts of CD8^+^ cells are reduced during the acute infection of FMDV as well (107). These results indicate that FMDV causes the dysregulation of T cells and B cells to guarantee the rapid replication of FMDV in the infected animals. Identification of the viral proteins that lead to the dysregulation of T cells and B cells will provide insights for modification of FMDV vaccine strain using a reverse genetic system.

### Suppression of Antiviral Immune Response by FMDV Virulence Factors

FMDV has a short replication cycle in its host, and its virulence factors alter the host cell environment to promote viral replication by inactivating host factors and blocking their functions (108). A number of viral proteins of FMDV have been demonstrated to play suppressive roles on host immune response that ensures the replication of the virus in the host (9, 10). These virulence factors inhibit host antiviral immune response by multiple manners which mainly include: suppression of synthesis of host proteins, blocking the synthesis of IFNs, and reducing the expression of IFN-stimulated genes (ISGs) as well as proinflammatory cytokines. Such as, overexpression of VP1 suppresses type-I IFN production and type I IFN response (109–111). Mutation of the crucial site 83E to 83K in FMDV VP1 impairs the interaction between VP1 and innate immune adaptor molecule MAVS, thereby decreasing the pathogenicity of the virus in pigs, which could be utilized for future development of FMDV vaccines (110). Together, mutation of the immunosuppressive domain or critical sites in the viral genome of vaccine strain is a prominent strategy and a rational approach to virus attenuation during preparation of future FMD vaccines.

#### Inhibition of Host Proteins Synthesis

FMDV inhibits the synthesis of host proteins in many ways, among which L^pro^ and 3C^pro^ play the most important functions (112). L^pro^ is a papain-like cysteine proteinase, which self-cleaves from the nascent viral polyprotein precursor, and plays a significant role in suppressing the innate immune response (113). Eukaryotic translation initiation factor 4 γ (eIF4G) is cleaved by L^pro^, leading to the suspension of host cap-dependent mRNA translation, thus shutting off host cell protein synthesis and allowing the virus to use the host cell protein synthesis machinery (cap-independent manner) (114). Moreover, L^pro^ cleaves host proteins such as nuclear factor kappa B (NF-κB), poly(A)-binding protein (PABP), polypyrimidine tract-binding protein (PTB), Gemin5, eIF3a, and eIF3b to inhibit the transcription system and cause cytopathic effect (CPE) (115). Although L^pro^ has strong function to block host antiviral response in various cell system, the function of L^pro^ in the host should be further investigated.

As another critical virulence factor, 3C^pro^ has the function of proteolytic enzymes, removing 20 amino acid residues at the N-terminal of host histone H3, thereby altering the transcription of chromosomes and blocking host translation (116). In addition, 3C^pro^ acts as an RNA helicase and cleaves eIF4A which is a part of the cap structure complex. 3C^pro^ also cleaves eIF4G in the late stage during viral infection, and the cleavage site targeted by 3C^pro^ is different with that targeted by L^pro^ (117). The cleavage of these factors results in the decreased host proteins synthesis rate which efficiently promotes FMDV replication. Design of inhibitors of picornaviral proteases has been carried out to develop potential drugs for treatment of picornavirus infection (118–120). The screening of inhibitors that target the proteinase of 3C^pro^ and L^pro^ might a potential strategy to eliminate the picornavirus from host (121–123).

#### Blocking the Synthesis of IFNs and Reducing the Expression of ISGs

**Figure 3:**
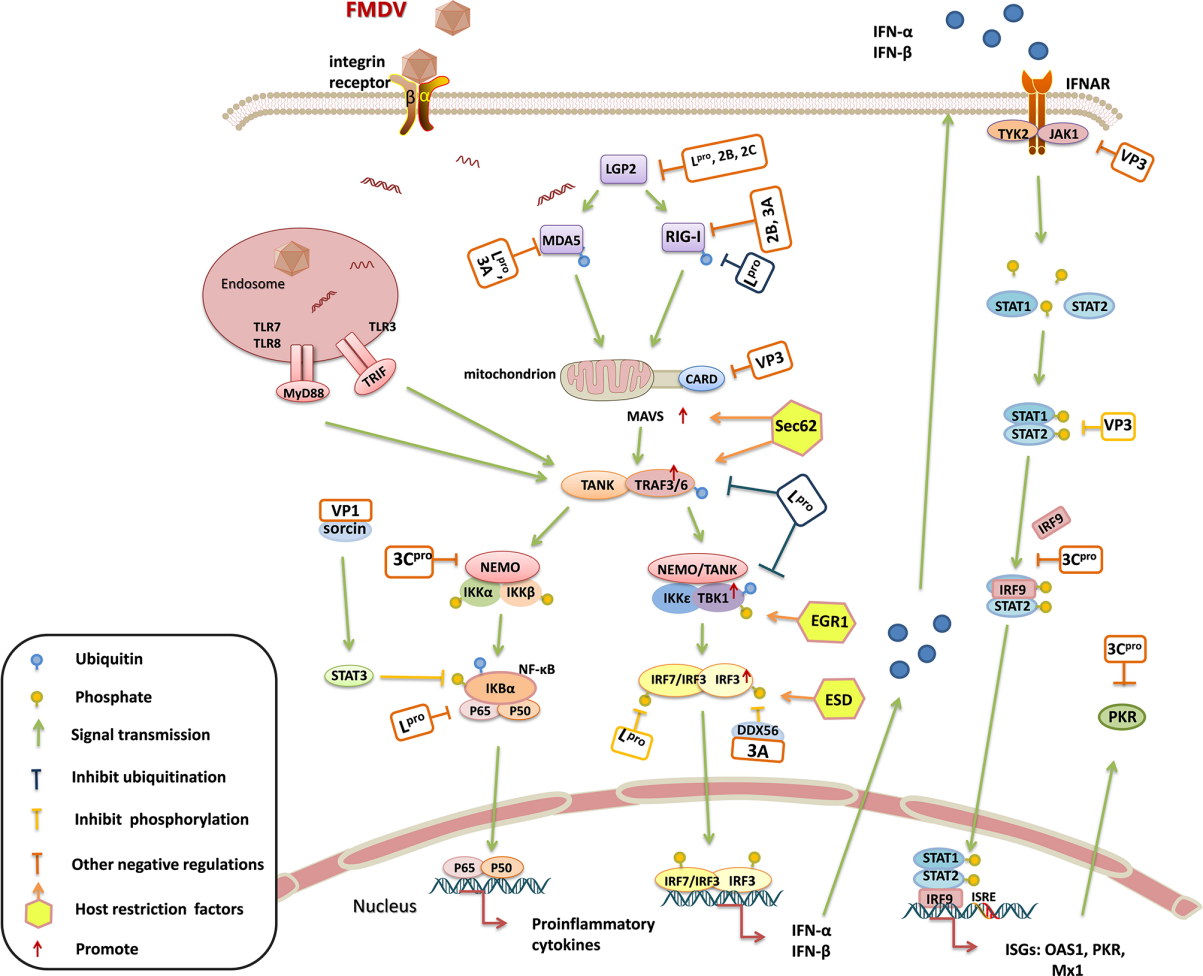
Schematic representation showing the interaction between foot-and-mouth disease virus (FMDV) viral proteins and host proteins in the innate immune system. FMDV invades into host cells through integrin receptors, the viral cytosolic RNAs and endosomal RNAs are recognized by RIG-I-like receptors (RLRs) [retinoic acid-inducible gene-I (RIG-I) and melanoma differentiation-associated gene 5 (MDA5] and Toll-like receptors (TLRs) (TLR3 and TLR7/8), triggering a signaling cascade, and inducing the production of interferons (IFNs) and cytokines to initiate direct or indirect antiviral responses. RIG-I and MDA-5 expose their CARD domains and then interact with the CARD of mitochondrial antiviral signaling protein (MAVS), while TLR3 and TLR7/8 interact with TRIF and MyD88, respectively. The recruited molecules TANK and TRAF3/6 subsequently activate different pathways through recruiting the IKK-α/β or TBK1/IKK-ϵ. IKK-α/β activates IκBα by phosphorylating it, and the phosphorylated IκBα is ubiquitinated and degraded by the proteasomes, allowing the release of p50/p65. The released p50/p65 translocates to the nucleus and binds to the specific gene promoters, initiating the expression of various proinflammatory cytokines. TBK1 phosphorylates IRF3 or IRF7, and induces the IRF3-IRF3 or IRF3-IRF7 dimerization. The IRF3-IRF3 or IRF3-IRF7 dimers transport to the nucleus, then bind to the IFNs promoters and initiate IFNs secretion. The synthesized IFNs bind to their specific receptors on the cell surface, activating TYK2 and JAK1 to induce STAT1/2 forming the phosphorylated heterodimers, which then interact with IRF9 and serves as a transcription factor complex (also known as ISGF3). The ISGF3 enters the nucleus and induces the expression of ISGs. Multiple virulence factors of FMDV target the components of the host innate immune system, thereby inhibiting the host antiviral responses. L^pro^, as a multifunctional protein, cleaves LGP2, acts as a deubiquitinating enzyme (DUB) to inhibit the ubiquitination of RIG-I, TRAF3/6, and TBK1, decreases the accumulation of p65, and hinders IRF3/7 phosphorylation. 3C^pro^, as an important virulent factor as well, cleaves NEMO, inhibits the binding of STAT1/2 to IRF9, induces the degradation of PKR through the lysosomal pathway. 3A interacts with DDX56 to inhibit the phosphorylation of IRF3. VP1 interacts with sorcin, activating STAT3 to inhibit IκBα phosphorylation. VP3 inhibits the expression of MAVS, and interacts with JAK1 to inhibit STAT1/2 phosphorylation and dimerization. In contrast, several host restriction factors also interact with the components in the pathways to enhance host antiviral response and suppress FMDV replication. Sec62 interacts with MAVS and TRAF3 to stabilize their status. EGR1 and ESD promote the phosphorylation of TBK1 and IRF3, respectively, to enhance type I IFN production and IFN response.

IFNs are a class of soluble glycoproteins secreted by host cells induced by virus infection or other stimulus, which mainly include IFN-α and IFN-β (type I IFN), IFN-γ (type II IFN), and IFN-λ (type III). ISGs are a group of antiviral genes that regulated by IFNs, the protein products of ISGs directly or indirectly suppress virus propagation at different stages in viral replication cycle (124). IFNs play a crucial role in both innate and adaptive immunity against virus infections. IFNs bind to their specific receptors on the cell membrane, and then trigger cascades of signal transduction to regulate the expression of ISGs that finally perform the antiviral function (125). PRRs are responsible for sensing the invading virus and induce IFNs production. PRRs mainly include Toll-like receptors (TLRs), RIG-I-like receptors (RLRs), NOD-like receptors (NLRs) and C-type lectin receptors (CLRs) (72). The RLRs containing retinoic acid-inducible gene-I (RIG-I), melanoma differentiation-associated gene 5 (MDA5) and Laboratory of Genetics and Physiology 2 (LGP2), the TLRs containing the TLR3, TLR7, and TLR8, and the NLR containing the NOD2 (73), have been reported to participate in the induction of immune responses during FMDV infection (**Figure 3**). These PRRs pathways consist of a complicated immune regulation network to be co-operative that constrains FMDV replication. Therefore, diverse mechanisms about the virulence factors to evade the immune network have been identified. The expression levels of various viral proteins are different in FMDV-infected cells. Therefore, the pathways are inhibited by the viral proteins to different extents. The host also has some compensation mechanisms to defend FMDV infection. A complicated progress is involved in this battle between host and FMDV.

L^pro^ plays multiple functions during counteraction of host immune response. L^pro^ inhibits the expression and phosphorylation of IFN regulatory factor (IRF) 3/7 to decrease type I IFN production (126). L^pro^ negatively regulates the expression level of IFN-λ1 (127). L^pro^ also acts as a deubiquitinating enzyme (DUB) that inhibits the ubiquitination of RIG-I, TBK1, TRAF3, and TRAF6 to suppress the signal transduction of RLR pathway (128). In addition, L^pro^ binds to host transcription factor ADNP (activity dependent neuroprotective protein) to interfere with its transcriptional function, which also leads to the decreased expression of IFNs and ISGs (129). The new targets attached by L^pro^ should be investigated, and the inhibitory sites in L^pro^ should also be identified, which will help to reveal the detailed mechanisms of FMD pathogenesis.

Both the L^pro^ and 3C^pro^ of FMDV counteract NF-κB activity, but they suppress NF-κB pathway activation through different mechanisms. L^pro^ cleaves the p65 subunit of NF-κB (RelA), leading to the reduction of NF-κB (130). 3C^pro^ specifically targets the Gln383 residue of NEMO to remove its C-terminal zinc finger structure domain, thereby blocking the activation of NF-κB and restrain its nuclear translocation (131). Although distinctive mechanisms are used by L^pro^ and 3C^pro^, the expression of various proinflammatory cytokines are inhibited by both L^pro^ and 3C^pro^, which proficiently promotes FMDV replication. 3C^pro^ also uses some unique mechanisms to inhibit IFN-induced antiviral effect. 3C^pro^ blocks the nuclear translocation of STAT1/STAT2 to block the activation of JAK-STAT signaling, thus inhibiting the expression of ISGs and counteracts host antiviral responses (132). Moreover, 3C^pro^ induces the degradation of PKR through the lysosomal pathway, thus overcoming the antiviral effect mediated by PKR (133). However, how does 3C^pro^ manipulate the lysosomal system to degrade PKR remains unknown. The direct suppressive effect of 3C^pro^ on other ISGs might also occur during FMDV replication, which is a strategy used by FMDV to impair host antiviral response.

FMDV 2B and 3A also negatively regulate the RLR-mediated IFN-β production. Both 2B and 3A interact with the immune sensors of RLRs pathway (134–136). Moreover, FMDV 3A could interact with host DDX56 protein to inhibit the production of type I IFN (137). A recent study shows that host cyclophilin A (CypA) protein degrades FMDV L^pro^ and 3A which rescues type I IFN production, however, FMDV 2B protein can interact with CypA to restrain the antiviral function of CypA, revealing a novel mechanism of 2B to block type I IFN production (138). This suggested a mutual interaction between host and virus. A complicated network exists between host and virus. The viral proteins and host proteins are the nodes in the protein-protein interaction network.

In addition to the non-structural proteins, the structural proteins of FMDV also antagonize host innate immune response. FMDV VP1 interacts with soluble resistance-related calcium binding protein (sorcin) to inhibit the production of type I IFN through negative regulation of NF-кB (139). FMDV VP3 interacts with the virus-induced signal adapter, mitochondrial antiviral signaling protein (MAVS), and inhibits its expression by interfering with its mRNA synthesis, thereby inhibiting the activation of RLRs pathway and type I IFN production (140). VP3 also inhibits the type II IFN response by interacting with JAK1/2 to suppress the phosphorylation and dimerization of the downstream signal molecule STAT1/2, thus blocking the nuclear translocation of STAT1/2 and ISGs expression. Meanwhile, VP3 destroys the assembly of JAK1 complex and degrades JAK1 through the lysosomal pathway (88). p53 induces the expression of ISGs such as ISG20, IRF9, RIG-I, and ISG15, thus playing an important role in antiviral innate immunity. Nucleoside diphosphate kinase 1 (NME1) is an activator of p53, but the VP4 of FMDV induces NME1 degradation through the lysosomal pathway. Therefore, FMDV VP4 protein plays an important role in inhibiting ISGs expression by regulation of p53 pathway signaling (141). The structural proteins are the main components of inactivated FMDV vaccines, elimination of the immunosuppressive sites in the structural proteins will enhance the efficiency of the vaccine and accelerate adaptive immune response.

#### Other Antagonistic Mechanisms Used by FMDV Virulence Factors

LGP2 is a homologous gene of RIG-I and MDA5 lacking of caspase activation and recruitment domain, it is considered that LGP2 performs different function comparing with RIG-I and MDA5 (142). LGP2 might play an important role in the regulation of RIG-I and MDA5-mediated antiviral function (143). FMDV L^pro^ interacts with LGP2, and cleaves its RGRAR amino acid sequence, thereby inhibiting LGP2-related antiviral activity (89). 3C^pro^ and 2B proteins also antagonize the antiviral effect mediated by LGP2 (144). NOD2 is one of NLR that inhibits the replication of FMDV. FMDV 2B, 2C, and 3C^pro^ can reduce the expression of NOD2 protein through different mechanisms, thus evading NOD2-mediated antiviral responses (145). FMDV infection manipulates mitogen-activated protein kinase (MAPK) signal pathway in favor of viral replication. While host ribosomal protein SA (RPSA) plays an important role in prevention of MAPK pathway activation and suppresses FMDV replication. To resist RPSA-mediated antiviral effect, FMDV VP1 interacts with RPSA and reactivates MAPK pathway signaling, thus promoting virus replication (146). FMDV utilizes the MAPK pathway, and the inhibitors targeting the MAPK pathway suppresses FMDV replication (146). Therefore, screening the inhibitors which regulate the pathways manipulated by FMDV might be helpful for developing antiviral drugs against FMDV.

Stress granules (SGs) are involved in the antiviral process during the replication of several viruses (147). A number of nucleating factors and messenger ribonucleoproteins (mRNPs) have been identified in the SGs (148). The nucleating factors Ras GTPase-activating protein-binding proteins 1 and 2 (G3BP1 and G3BP2) and Src-associated protein of 68 kDa (Sam68) have been found to be involved in suppression of FMDV replication. G3BP1 enhances the expression of RIG-I and MAD5, so it is considered as an important antiviral protein in the host. Leucine rich repeat-containing 25 (LRRC25) is an autophagy-related protein, and FMDV 3A induces the reduction of G3BP1 by up-regulation of the expression of LRRC25, thus inhibiting G3BP1-mediated antiviral function (149). G3BP are also used as the scaffolding proteins of SGs, L^pro^ and 3C^pro^ can target G3BP and antagonize the antiviral effect of SGs. L^pro^ cleaves both G3BP1 and G3BP2, while 3C^pro^ cleaves G3BP1. Ectopic expression of G3BP1 inhibited FMDV IRES activity and suppress FMDV replication. 3C^pro^ cleaves G3BP1 to enhance viral translation and promote FMDV replication (150, 151). FMDV triggers SGs formation early during infection (150). SGs are associated with innate immune response and are potential platforms that activate or amplify downstream innate immune signaling (152–154). Whether the viral proteins also suppress the activation of the innate immune signaling in SGs during FMDV infection remains unknown. The viral proteins wrapped in the SGs should be determined. Meanwhile, whether some viral proteins perform functions to disassemble SGs should also be explored. Sam68 binds to the IRES motif of FMDV and interferes with the translation of viral RNA, the 3C^pro^ and 3D^pol^ interact with Sam68 and prevent the binding of Sam68 with the IRES that leads to the rapid replication of FMDV (155). In addition, Sam68 is a regulator of TLR signaling (156). The 3C^pro^ and 3D^pol^ might also interfere with the TLR signaling during FMDV infection. These results suggest that multiple viral proteins are involved in antagonize host antiviral response, and different mechanisms have been employed by various viral proteins. The coordination among various viral proteins might determine the outcome of viral infection.

## Autophagy, Apoptosis, and Golgi-Endoplasmic Reticulum Pathway in FMDV-Infected Cells

Autophagy is a lysosome-dependent degradation pathway existing in eukaryotes (157, 158). The viral infection or several other stimuli can induce the formation of autophagosomes, and the autophagosomes then fuse with lysosomes, leading to the formation of autolysosomes (159). The acidic environment in the autolysosomes gives rise to the activation of the enzymes essential to degrade aggregate-prone or misfolded proteins, dysfunctional or surplus organelles, and the invaded pathogens (160). It is suggested that FMDV promotes the autophagy in the early stage in order to help viral infection. HSPB1 (heat shock protein beta-1) is crucial for formation of the autophagosomes (161). The mammalian target of rapamycin (mTOR) inhibits the cellular autophagy (162). FMDV VP2 interacts with HSPB1 to activate EIF2S1-ATF4 signal pathway and inhibits AKT-MTOR pathway, thus inducing autophagy and promoting viral replication (163). Japanese encephalitis virus infection causes autophagy to promote the traffic of virus to autophagosomes for subsequent steps of infection (164). FMDV might manipulate the autophagosomes to establish a good environment for viral infection. In addition, FMDV induces the redistribution of LC3 to colocalize with the autophagy protein ATG5. FMDV VP1 also colocalizes with LC3 to form the LC3 punctate, then the autophagy is induced which helps FMDV replication (165). PKR-like ER kinase (PERK) pathway is an important pathway involving regulation of autophagy. A recent study indicates that FMDV infection induces ER stress and unfolded protein response (UPR) through PERK-mediated pathway, which in turn inducing autophagy. The inhibition of PERK pathway blocks autophagy and decreases the expression levels of IFN-β and IFN-λ3, which inhibits FMDV replication. Therefore, FMDV-induced autophagy promotes the multiplication of the virus by activation of PERK pathway (166). This suggested that multiple viral proteins and multiple mechanisms are involved in manipulation of autophagy during FMDV infection.

Although the involvement of autophagy in positive regulation of FMDV replication has been reported, the mechanism of autophagy in FMDV replication remains poorly understood. Besides, several controversial studies also reported that FMDV infection induces autophagy but does not promote the viral replication or even suppresses FMDV replication (108, 167, 168). A recent study by Han et al. suggests that FMDV infection induces the activation of PERK and ATF6 mediated UPR but does not influence the replication of FMDV (168), and their another study indicates that the autophagy-related protein ATG5-ATG12 conjugate plays an important role in autophagy showing an antiviral effect (169). ATG5-ATG12 conjugate positively regulates NF-кB signal pathway by promoting IKKα/β phosphorylation, accelerating IκBα degradation, and inhibiting p65 degradation (170). Meanwhile, ATG5-ATG12 enhances the phosphorylation of TBK1 and IRF3, thus promoting the activation of IRF3-mediated type I IFN signal pathway. FMDV 3C^pro^ is identified to inhibit the host antiviral response by degradation of ATG5-ATG12 and suppress the occurrence of autophagy (169). The regulatory roles of autophagy in FMDV-infected cells identified in their studies are contradictory to the previous reports. The different virus strains or cell types used in the experiments might cause this difference. The association between autophagy and FMDV replication in animals at different infection stage should be further investigated to solve this confusion.

Microtubule-associated protein light-chain kinase 3 (LC3) is a component of the mature autophagosome membrane, which is used as a marker of autophagosomes (171). The GFP-labeled LC3 molecule can be redistributed and co-locates with viral non-structural proteins 2B, 2C, and 3A in FMDV-infected cells (172). The 2B, 2C, and 3A proteins promote the membrane rearrangement to form the replication complexes for FMDV, providing replication sites for the virus in cells. 2B protein inserts itself into the endoplasmic reticulum (ER), acting as an ion channel protein that forms a hole in the ER (172). This pore increases the permeability of the cell membrane and damages the homeostasis of Ca^2+^. Therefore, it not only causes the membrane damage which helps the release of viral offspring, but also blocks the secretion of host proteins (173). For FMDV 2C protein, host protein Beclin1 is a ligand of 2C, the interaction between Beclin1 and 2C prevents the fusion of autophagosomes and lysosomes to inhibit autophagy (174). Another ligand of 2C, Vimentin, also interacts with 2C. The 2C-vimentin interaction may modulate the host cell environment to allow for rapid viral replication (175). FMDV 2C also disrupts the Golgi-ER secretory pathway to inhibit the transportation of host protein from the Golgi apparatus to cell surface and induce autophagy in favor of viral replication (176). These viral proteins might reorganize the intracellular compartments to build virus factories to benefit viral replication in a changed subcellular microenvironment.

In addition to inducing immune responses, inflammatory responses and autophagy, infection by FMDV triggers apoptosis of virus-infected cells (177). Apoptosis is a form of programmed cell death that maintains the stable internal environment of the host. FMDV infection and replication are highly associated with apoptosis (108). Extensive cellular apoptosis can be induced after FMDV infection, which plays a role in the outcome of FMDV infection (178). The RGD motif of FMDV VP1 interacts with the integrin receptor, which activates the caspases 3, 8, and 9, and the expression of Bcl 2 is then down-regulated and leads to the release of cytochrome C (Cyt-C) from mitochondria into the cytosol. The released Cyt-C in turn induces a series of biochemical reactions that results in the apoptosis (179). The N-myc and STAT interactor (Nmi) protein negatively regulates the virus-induced type I IFN production (180, 181). FMDV 2C interacts with Nmi and induces apoptosis to promote the viral replication (182). Poly (rC) binding protein 2 (PCBP2) interacts with FMDV VP0 protein to promote the degradation of MAVS *via* activation of the apoptotic pathway, which increases the replication of FMDV (183). FMDV use apoptosis as a mechanism of cell killing and virus spread. The viral proteins block or delay apoptosis by protein-protein interaction to ensure the production of progeny. The interaction between viral protein and host protein is crucial for subverting host cell defense systems to ensure viral survival, replication and proliferation (108). Investigation of the mechanisms of FMDV to regulate apoptosis could offer insights into how this knowledge may be used for future research and FMD control.

**Figure 4:**
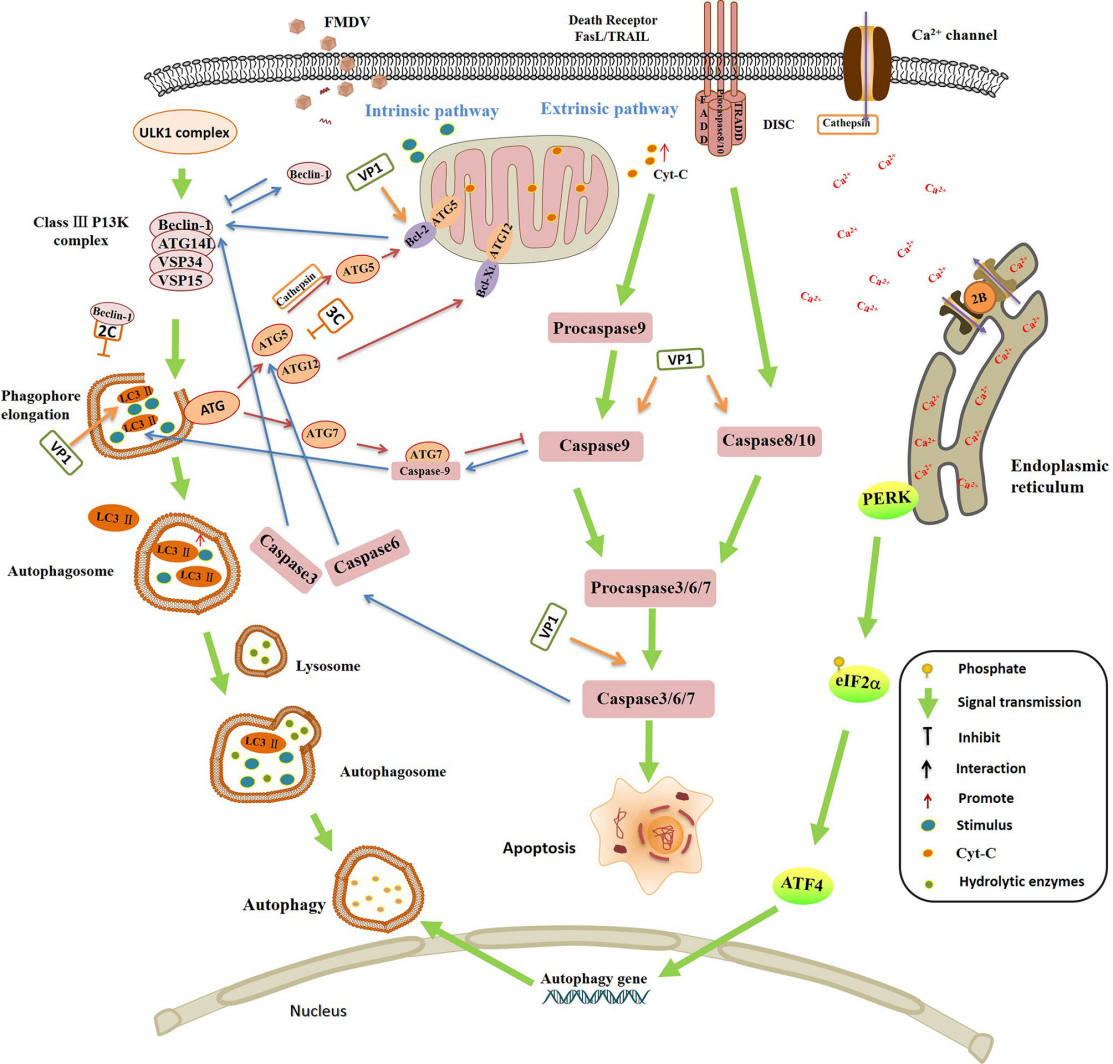
The interplay between autophagy and apoptosis, and the regulation of autophagy and apoptosis by foot-and-mouth disease virus (FMDV) infection. Autophagy and apoptosis are essential physiological responses to maintain cell and body homeostasis. The relationship between autophagy and apoptosis is complicated under various stimulus, they promote the activation of each other in some conditions, but also inhibit each other in in some special conditions. Bcl-2 binds to Beclin-1 and separates Beclin-1 from Class III P13K complex, leading to the inhibition of autophagy. Caspase-3 inhibits autophagy and activates apoptosis by cleavage of Beclin-1. Caspase-6 disrupts autophagy by induction of ATG5 and Beclin-1 cleavage. The interaction between caspase-9 and ATG7 promotes the formation of LC3-II and activation of autophagy, but inhibits the caspase-9 apoptotic activity. The cleaved ATG5 catalyzed by cathepsin migrates from cytoplasm to mitochondria, and then interacts with Bcl-XL to promote the release of Ccyt-C and activation of caspases. Uncoupled ATG12 positively regulates mitochondrial apoptosis by binding to Bcl-2 and inhibits the function of Bcl-2. ATG12 promotes the up-regulation of Bax and enhances the release of Cyt-C. FMDV infection is highly associated with autophagy and apoptosis. FMDV VP1 interacts with the integrin receptor, which activates the caspases 3, 8 and 9, and down-regulates the expression of Bcl 2, leading to the release of Cyt-C, and finally induces the apoptosis. VP1 colocalizes with LC3 to induce the formation of LC3 punctate, contributing to autophagy and promoting FMDV replication. FMDV 3C^pro^ degrades ATG5-ATG12 and suppresses the occurrence of autophagy. FMDV activates PKR-like ER kinase (PERK) pathway and promotes the phosphorylation of eIF2α, and then induces the expression of autophagy gene, resulting in the activation of autophagy that promotes the multiplication of FMDV. The interaction between Beclin-1 and 2C prevents the fusion of autophagosomes with lysosomes which leads to the inhibition of autophagy. FMDV 2B protein inserts itself into the endoplasmic reticulum (ER), acting as an ion channel protein that forms a hole in the ER, which damages the homeostasis of Ca^2+^ and affects the mature of autophagosome membrane.

Autophagy and apoptosis are essential physiological mechanisms to maintain cell and body homeostasis. At present, it is found that autophagy and apoptosis may have the following three relationships: 1) apoptosis and autophagy promote the activation of each other; 2) autophagy is a necessary condition for apoptosis, inhibition of autophagy can delay the occurrence of apoptosis; and 3) apoptosis and autophagy antagonize each other. The crosstalk between autophagy and apoptosis depends on the interaction between the key proteins in the involved pathways (184, 185). Bcl-2 family proteins inhibit mitochondrial release of Cyt-C, which plays a key role in regulation of apoptosis. Beclin 1 is a component of Class III P13K complex, and it is necessary for the formation of autophagosome. Bcl-2 binds to Beclin-1 and separates Beclin 1 from Class III P13K complex, leading to the inhibition of autophagy (186). ATG5 and ATG12 are necessary to induce autophagy. But interestingly, the unconjugated forms of ATG5 and ATG12 have the effect of inducing apoptosis. In the induction of apoptosis, the cleaved ATG5 catalyzed by cathepsin can migrate from cytoplasm to mitochondria, and then interacts with Bcl-X_L_ to promote the release of Ccyt-C and activation of caspases (187). Uncoupled ATG12 positively regulates mitochondrial apoptosis by binding to Bcl-2 and inhibits the function of Bcl-2. ATG12 up-regulates of Bax and enhances the release of Cyt-C as well (188). Caspase-3 inhibits autophagy and activates apoptosis by cleaving Beclin-1 (189). The activated caspase-6 suppresses autophagy by cleavage of two key autophagy regulators, ATG5 and Beclin-1. The interaction between caspase-9 and ATG7 promotes the formation of LC3-II and activation of autophagy, while, ATG7 inhibits the caspase-9 apoptotic activity and this is not related to the autophagic function of ATG7. ATG7-caspase-9 complex has a dual function in regulation of apoptosis and autophagy (190). The complicated link and regulation between autophagy and apoptosis under different environment might have resulted in the ambiguous reports on the role of autophagy during FMDV infection (**Figure 4**).

## Host Restriction Factors and Their Functions During FMDV Replication

In the virus infected cells, the viral proteins or genomes interact with a large number of host factors that facilitate or hinder viral replication (191). The host restriction factors are host cellular proteins that interfere with the different stages of the viral life cycle contributing to the defense against viral infections (192, 193). A lot of host restriction factors that limit FMDV replication by enhancing the IFNs, proinflammatory cytokines production or ISGs expression, have been identified.

Early growth response gene-1 (EGR1), also known as zif268, is a host transcription regulator involved in the activation of multiple pathways signal transduction (194, 195). As a zinc finger DNA binding protein, EGR1 binds to the target gene promoter sequence to regulate the expression of a variety of gene families (196). In addition, it mediates the signal transduction cascade in cell differentiation, apoptosis and proliferation (197). Both the transcripts and protein levels of EGR1 are up-regulated in FMDV-infected cells. Overexpression of EGR1 inhibits FMDV replication, and knockdown of EGR1 expression promotes FMDV replication, suggesting an antiviral role of EGR1 against FMDV. It is demonstrated that EGR1 promotes type I IFN production by increasing the phosphorylation of TBK1, which suppresses FMDV replication (198). Enhancing the function of positive regulatory factors in antiviral system in cells might be a strategy to restrict FMDV replication.

CypA is also a host restriction factor targeting FMDV (138). It is one of the main members of the PPIase family, which catalyzes the cis-trans isomerization of the peptide-proline bonds, and is crucial for the normal physiological activities of the host (199, 200). CypA is initially identified as the primary target of the immunosuppressive drug cyclosporine A (CsA), which acts as an immunophilin to block the activation of mammalian T cells by forming CsA-CypA complex (201). In addition, CypA regulates NF-ĸB activity, activates transcription 3 (Stat3), and facilitates IL-6-induced signal transduction (202, 203). As mentioned above, CypA reduces the expression level of FMDV L^pro^ through the proteasome pathway, then improving the integrity of host eIF4G, restoring the synthesis of host proteins. Meanwhile, CypA inhibits the expression 3A protein, which neutralizes 3A-mediated antagonistic role on type I IFNs production. On the contrary, FMDV 2B protein interacts with CypA and antagonizes the functions of CypA to maintain FMDV replication in the cells (138). CypA has also be reported to promote the replication of another picornavirus EV71 (204), suggesting that it is critical for suppression of picornaviruses replication. CypA also inhibits influenza virus replication by degradation of the M1 protein *via* the proteasome-dependent pathway (205). How CypA regulates the proteasome system to decrease the expression of viral proteins remains unknown. The CypA inducer might be an effective drug to restrict FMDV replication in host cells.

Esterase D (ESD) is an S-formylglutathione hydrolase with the serine hydrolase activity (206). It is considered to be related to the retinoblastoma disease, and if the activity of ESD enzyme decreases, people are prone to having the diseases (207). ESD mRNA is significantly up-regulated in FMDV-infected cells. It shows that ESD can regulate type I IFN production by promoting the phosphorylation of IRF3, and the upregulation of ESD positively regulates ISG expression (208). Therefore, ESD is an important host restriction factor that inhibits the replication of FMDV.

Heat shock proteins (Hsps) are found to be involved in many stages of viral replication cycle (209). Hsps promote protein folding and transportation, and prevent protein damage caused by heat and other stresses (210). DNAJA3 is a member of the DnaJ heat shock protein family (Hsp40), which interacts with FMDV VP1 and degrades VP1 through the lysosomal pathway. FMDV VP1 suppresses type I IFN response. The degradation of VP1 by DNAJA3 therefore restores type I IFN response and inhibits FMDV replication (111). DNAJA3 is predominantly localized in mitochondrial matrix, MAVS also distributes in mitochondria. Suppression of DnaJA3 induces mitochondrial fragmentation in HeLa cells (211). Whether DNAJA3 also enhances type I IFN production by regulation of MAVS function or mitochondrial homeostasis should be investigated.

Translocation protein Sec62 is a membrane protein located in ER, which acts as an autophagy receptor and transfers specific components to autophagosome-lysosome for degradation (212). Sec62 is an important molecule to maintain the cell homeostasis (213). IRE1α (Inositol Requiring kinase Enzyme 1 alpha) is also a transmembrane protein localized to the ER, which can activate RIG-I and its downstream effector molecules to play an antiviral role. Sec62 promotes IRE1α phosphorylation and activate IRE1α-RIG-I pathway by regulation of TRAF3 function, which promotes type I IFN production and suppresses FMDV replication. More and more host restriction factors are identified to play antiviral effect during FMDV infection. How do the viral factors counteract the antiviral functions of these factors should be exploited in future to elucidate the pathogenesis of FMDV. In addition, development of the agonists of host restriction factors might be also a prominent strategy to restrict FMDV replication.

## Concluding Remarks

In this review, we summarized the pathogenesis of FMD, FMDV receptors and cell tropism, innate/adaptive immune system dysfunction, autophagy, apoptosis, and Golgi-endoplasmic reticulum pathways in FMDV infection to describe the gap in the related knowledge. Meanwhile, we summarized how host defends FMDV infection through various host restriction factors. The dysregulation of host antiviral system by the virus has contributed to serious pathogenesis. There remain many knowledge gaps to be further explored and investigated, especially the regulation of the virus in different host at different infection stage. The single-cell analysis of the infected cells and the involved signaling during FMDV infection in its natural hosts is a prominent way to uncover more pathogenic mechanisms of FMD. The third groups of receptors of FMDV remain unidentified. The mechanisms of how the dysregulation of the functions of immune cells, cellular biological responses, and pathway activation contribute to pathogenesis is still largely elusive. Moreover, the *in vivo* relevance of many observations coming from the overexpression of viral proteins in various cell systems remains to be confirmed. FMDV infection induces high levels of inflammatory cytokines, the inhibitors of several critical pathogenic cytokines might be potential drugs against FMDV infection. Therefore, the identification of critical host factors involving in pathogenesis of FMDV is extremely important. Further understanding these insights will help to clarify the detailed mechanisms of causing FMD. We hope that this review will enhance our understanding of the virus-host interactions during FMDV infection, and provide insights for the development of high-efficient vaccines or antivirals against FMDV and other picornaviruses.

## Author Contributions

KL, ZZ, and HZ conceptualized the review and wrote the manuscript. CW edited the manuscript and conceived the figures. FY and WC collected data. All authors contributed to the article and approved the submitted version.

## Funding

This work was supported by grants from the National Key Research and Development Project (2017YFD0501103 and 2017YFD0501800), the Key Technologies R&D Program of Gansu Province (19ZDNA001), the Key Development and Research Foundation of Yunnan (2018BB004), and the Chinese Academy of Agricultural Science and Technology Innovation Project (CAAS-ASTIP-2020-LVRI and CAAS-ZDRW202006).

## Conflict of Interest

The authors declare that the research was conducted in the absence of any commercial or financial relationships that could be construed as a potential conflict of interest.
